# Supplementary material for: Adjunctive phage therapy improves antibiotic treatment of ventilator-associated-pneumonia with Pseudomonas aeruginosa
Source: Nat Commun. 2025 May 15;16:4500. doi: 10.1038/s41467-025-59806-y (PMC12078490; doi:10.1038/s41467-025-59806-y)
Supplement: Supplementary file 1 — Supplementary Information [file 41467_2025_59806_MOESM1_ESM.pdf]

Supplementary Information

Adjunctive phage therapy improves antibiotic treatment of Ventilator-Associated-Pneumonia with *Pseudomonas aeruginosa*

Chantal Weissfuss[1,#], Jingjing Li[1], Ulrike Behrendt[1], Karen Hoffmann[1], Magdalena Bürkle[1], Chunjiang Tan[1], Gopinath Krishnamoorthy[1], Imke H. E. Korf[2], Christine Rohde[3], Baptiste Gaborieau[4,5,6], Laurent Debarbieux[4], Jean-Damien Ricard[5,6], Martin Witzenrath[1,7], Matthias Felten[1,*] and Geraldine Nouailles[1,#,*]

[1] Charité - Universitätsmedizin Berlin, Corporate Member of Freie Universität Berlin and Humboldt-Universität zu Berlin, Department of Infectious Diseases, Respiratory Medicine and Critical Care, Berlin, Germany.
[2] Pharmaceutical Biotechnology, Fraunhofer Institute for Toxicology and Experimental Medicine, Braunschweig, Germany.
[3] Leibniz Institute DSMZ-German Collection of Microorganisms and Cell Cultures, Braunschweig, Germany.
[4] Institut Pasteur, Université Paris Cité, CNRS UMR6047, Department of Microbiology, Bacteriophage Bacteria Host Laboratory, Paris, France
[5] Université Paris-Cité, Inserm, UMR 1137, Infection Antimicrobials Modelling Evolution, Paris, France.
[6] APHP, Hôpital Louis Mourier, DMU ESPRIT, Service de Médecine Intensive Réanimation, Colombes, France.
[7] German Center for Lung Research (DZL), Berlin, Germany
* Jointly supervised to this work.

[#] Correspondence should be addressed to chantal.weissfuss@charite.de (C.W.) and geraldine.nouailles@charite.de (G.N.)

Supplementary Information

Supplementary Table S1. Clinical disease score for murine VAP model.

Symptom	Score
**Fur**	
Shiny	0
Dishelved	1
**Eyes**	
Bright	0
Tearing	1
**Behaviour**	
Curious, awaken	0
Less active (cage-side observation)	1
Less reactive to external stimulus	2
**Breathing**	
Steady, consistent	0
Hyper- /hypoventilation	1
Labored	2
**Temperature p. i.**	
> 35 °C	0
≤ 35 °C	1
≤ 30 °C	2
**Body weight change p. i.**	
<5 %	0
>5 % ≤ 10 %	1
>10 % ≤ 20 %	2
**Total clinical score**	

p. i., post infection.

Supplementary Table S2. Summary of statistics (Figure 4b) from the bacterial load at 4 hpi from cyclic stretch experiments with the Flexcell® FX-5000™ Tension System. Data were determined by two-way ANOVA with Tukey´s multiple comparisons test; n = 3 with duplicates per sample per day. Combination ++++ (Meropenem 5 × MIC, Phages MOI 0.5); Combination +++ (Meropenem 0.5 × MIC, Phages MOI 0.5); Combination ++ (Meropenem 0.05 × MIC, Phages MOI 0.05); Combination + (Meropenem 0.005 × MIC, Phages MOI 0.005). Meropenem MIC = 1 µg/mL.

**Tukey's multiple comparisons test**	**Summary**	**Adjusted P Value**
4 hpi:Control vs. 4 hpi:Mero 5 x MIC	**	0,0014
4 hpi:Control vs. 4 hpi:Mero 0.5 x MIC	ns	0,5055
4 hpi:Control vs. 4 hpi:Phages MOI 0.5	****	<0,0001
4 hpi:Control vs. 4 hpi:Phages MOI 0.005	*	0,0279
4 hpi:Control vs. 4 hpi:Combination ++++	****	<0,0001
4 hpi:Control vs. 4 hpi:Combination +++	****	<0,0001
4 hpi:Control vs. 4 hpi:Combination ++	****	<0,0001
4 hpi:Control vs. 4 hpi:Combination +	**	0,0091
4 hpi:Mero 5 x MIC vs. 4 hpi:Mero 0.5 x MIC	ns	0,7914
4 hpi:Mero 5 x MIC vs. 4 hpi:Phages MOI 0.5	*	0,0202
4 hpi:Mero 5 x MIC vs. 4 hpi:Phages MOI 0.005	ns	>0,9999
4 hpi:Mero 5 x MIC vs. 4 hpi:Combination ++++	**	0,0021
4 hpi:Mero 5 x MIC vs. 4 hpi:Combination +++	**	0,0021
4 hpi:Mero 5 x MIC vs. 4 hpi:Combination ++	ns	0,8422
4 hpi:Mero 5 x MIC vs. 4 hpi:Combination +	ns	>0,9999
4 hpi:Mero 0.5 x MIC vs. 4 hpi:Phages MOI 0.5	****	<0,0001
4 hpi:Mero 0.5 x MIC vs. 4 hpi:Phages MOI 0.005	ns	0,98
4 hpi:Mero 0.5 x MIC vs. 4 hpi:Combination ++++	****	<0,0001
4 hpi:Mero 0.5 x MIC vs. 4 hpi:Combination +++	****	<0,0001
4 hpi:Mero 0.5 x MIC vs. 4 hpi:Combination ++	*	0,0169
4 hpi:Mero 0.5 x MIC vs. 4 hpi:Combination +	ns	0,979
4 hpi:Phages MOI 0.5 vs. 4 hpi:Phages MOI 0.005	*	0,0257
4 hpi:Phages MOI 0.5 vs. 4 hpi:Combination ++++	ns	>0,9999
4 hpi:Phages MOI 0.5 vs. 4 hpi:Combination +++	ns	>0,9999
4 hpi:Phages MOI 0.5 vs. 4 hpi:Combination ++	ns	0,8268
4 hpi:Phages MOI 0.5 vs. 4 hpi:Combination +	**	0,0042
4 hpi:Phages MOI 0.005 vs. 4 hpi:Combination ++++	**	0,0035
4 hpi:Phages MOI 0.005 vs. 4 hpi:Combination +++	**	0,0035
4 hpi:Phages MOI 0.005 vs. 4 hpi:Combination ++	ns	0,774
4 hpi:Phages MOI 0.005 vs. 4 hpi:Combination +	ns	>0,9999
4 hpi:Combination ++++ vs. 4 hpi:Combination +++	ns	>0,9999
4 hpi:Combination ++++ vs. 4 hpi:Combination ++	ns	0,3247
4 hpi:Combination ++++ vs. 4 hpi:Combination +	***	0,0004
4 hpi:Combination +++ vs. 4 hpi:Combination ++	ns	0,3247
4 hpi:Combination +++ vs. 4 hpi:Combination +	***	0,0004
4 hpi:Combination ++ vs. 4 hpi:Combination +	ns	0,4923

Supplementary Table S3. Summary of statistics (Figure 4b) from the bacterial load at 8 hpi from cyclic stretch experiments with the Flexcell® FX-5000™ Tension System. Data were determined by two-way ANOVA with Tukey´s multiple comparisons test; n = 3 with duplicates per sample per day. Combination ++++ (Meropenem 5 × MIC, Phages MOI 0.5); Combination +++ (Meropenem 0.5 × MIC, Phages MOI 0.5); Combination ++ (Meropenem 0.05 × MIC, Phages MOI 0.05); Combination + (Meropenem 0.005 × MIC, Phages MOI 0.005). Meropenem MIC = 1 µg/mL.

Tukey's multiple comparisons test	Summary	Adjusted P Value
8 hpi:Control vs. 8 hpi:Mero 5 x MIC	****	<0,0001
8 hpi:Control vs. 8 hpi:Mero 0.5 x MIC	****	<0,0001
8 hpi:Control vs. 8 hpi:Phages MOI 0.5	****	<0,0001
8 hpi:Control vs. 8 hpi:Phages MOI 0.005	****	<0,0001
8 hpi:Control vs. 8 hpi:Combination ++++	****	<0,0001
8 hpi:Control vs. 8 hpi:Combination +++	****	<0,0001
8 hpi:Control vs. 8 hpi:Combination ++	****	<0,0001
8 hpi:Control vs. 8 hpi:Combination +	****	<0,0001
8 hpi:Mero 5 x MIC vs. 8 hpi:Mero 0.5 x MIC	ns	0,9301
8 hpi:Mero 5 x MIC vs. 8 hpi:Phages MOI 0.5	ns	>0,9999
8 hpi:Mero 5 x MIC vs. 8 hpi:Phages MOI 0.005	ns	>0,9999
8 hpi:Mero 5 x MIC vs. 8 hpi:Combination ++++	ns	0,0655
8 hpi:Mero 5 x MIC vs. 8 hpi:Combination +++	ns	0,0655
8 hpi:Mero 5 x MIC vs. 8 hpi:Combination ++	ns	0,405
8 hpi:Mero 5 x MIC vs. 8 hpi:Combination +	ns	>0,9999
8 hpi:Mero 0.5 x MIC vs. 8 hpi:Phages MOI 0.5	ns	0,9985
8 hpi:Mero 0.5 x MIC vs. 8 hpi:Phages MOI 0.005	ns	0,9718
8 hpi:Mero 0.5 x MIC vs. 8 hpi:Combination ++++	***	0,0004
8 hpi:Mero 0.5 x MIC vs. 8 hpi:Combination +++	***	0,0004
8 hpi:Mero 0.5 x MIC vs. 8 hpi:Combination ++	**	0,0059
8 hpi:Mero 0.5 x MIC vs. 8 hpi:Combination +	ns	0,6616
8 hpi:Phages MOI 0.5 vs. 8 hpi:Phages MOI 0.005	ns	>0,9999
8 hpi:Phages MOI 0.5 vs. 8 hpi:Combination ++++	*	0,0142
8 hpi:Phages MOI 0.5 vs. 8 hpi:Combination +++	*	0,0142
8 hpi:Phages MOI 0.5 vs. 8 hpi:Combination ++	ns	0,1358
8 hpi:Phages MOI 0.5 vs. 8 hpi:Combination +	ns	0,9995
8 hpi:Phages MOI 0.005 vs. 8 hpi:Combination ++++	*	0,0412
8 hpi:Phages MOI 0.005 vs. 8 hpi:Combination +++	*	0,0412
8 hpi:Phages MOI 0.005 vs. 8 hpi:Combination ++	ns	0,2983
8 hpi:Phages MOI 0.005 vs. 8 hpi:Combination +	ns	>0,9999
8 hpi:Combination ++++ vs. 8 hpi:Combination +++	ns	>0,9999
8 hpi:Combination ++++ vs. 8 hpi:Combination ++	ns	>0,9999
8 hpi:Combination ++++ vs. 8 hpi:Combination +	ns	0,2095
8 hpi:Combination +++ vs. 8 hpi:Combination ++	ns	>0,9999
8 hpi:Combination +++ vs. 8 hpi:Combination +	ns	0,2095
8 hpi:Combination ++ vs. 8 hpi:Combination +	ns	0,7518

Supplementary Table S4. Summary of statistics (Figure 4b) from the bacterial load at 4 hpi vs. 8 hpi from cyclic stretch experiments with the Flexcell® FX-5000™ Tension System. Data were determined by two-way ANOVA with Tukey´s multiple comparisons test; n = 3 with duplicates per sample per day. Combination ++++ (Meropenem 5 × MIC, Phages MOI 0.5); Combination +++ (Meropenem 0.5 × MIC, Phages MOI 0.5); Combination ++ (Meropenem 0.05 × MIC, Phages MOI 0.05); Combination + (Meropenem 0.005 × MIC, Phages MOI 0.005). Meropenem MIC = 1 µg/mL.

Tukey's multiple comparisons test	Summary	Adjusted P Value
4 hpi:Control vs. 8 hpi:Control	**	0,0066
4 hpi:Mero 5 x MIC vs. 8 hpi:Mero 5 x MIC	ns	0,9978
4 hpi:Mero 0.5 x MIC vs. 8 hpi:Mero 0.5 x MIC	ns	0,9775
4 hpi:Phages MOI 0.5 vs. 8 hpi:Phages MOI 0.5	ns	0,1076
4 hpi:Phages MOI 0.005 vs. 8 hpi:Phages MOI 0.005	ns	0,9969
4 hpi:Combination ++++ vs. 8 hpi:Combination ++++	ns	>0,9999
4 hpi:Combination +++ vs. 8 hpi:Combination +++	ns	>0,9999
4 hpi:Combination ++ vs. 8 hpi:Combination ++	ns	0,8766
4 hpi:Combination + vs. 8 hpi:Combination +	ns	0,6538

Supplementary Information

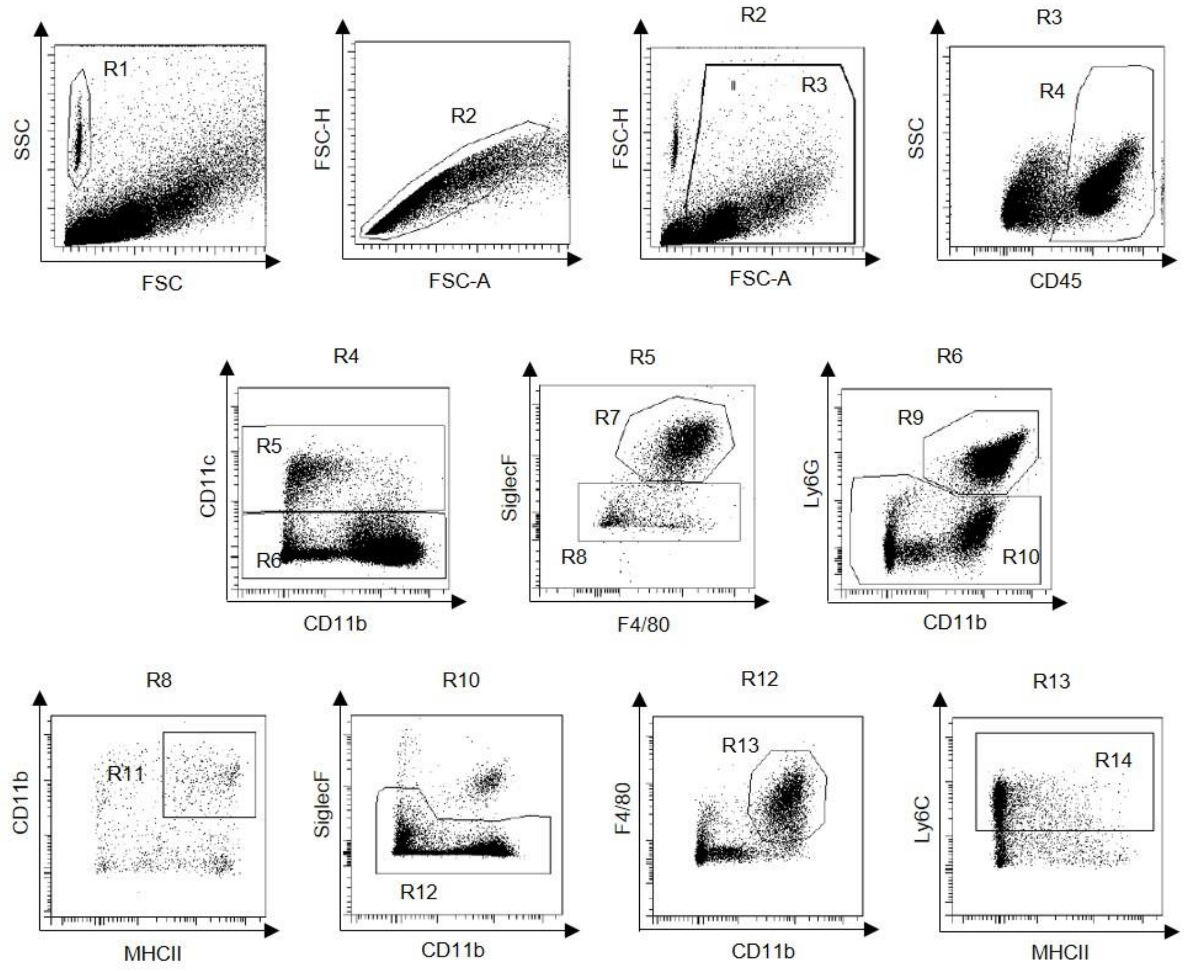

R1 beads
R2 singlets
R3 minus debris
R4 CD45+ cells (**leucocytes**)
R5 CD45$^+$ CD11c$^+$ cells
R7 CD45$^+$ CD11c$^+$ SiglecF$^+$ F4/80$^+$ (**alvM**)
R8 CD45$^+$ CD11c$^+$ SiglecF$^-$ F4/80$^-$ (DCs)
R11 CD45$^+$ CD11c$^+$ CD11b$^+$ SiglecF$^-$ F4/80$^-$ MHCII$^+$ (**matDCs**)
R6 CD45$^+$ CD11c$^-$ cells
R9 CD45$^+$ Ly6G$^-$ CD11b^{high} cells (**PMNs**)
R10 CD45$^+$ Ly6G$^-$ CD11c$^-$ cells
R12 CD45$^+$ Ly6G$^-$ CD11c$^-$ SiglecF$^-$ cells
R13 CD45$^+$ Ly6G$^-$ CD11c$^-$ SiglecF$^-$ CD11d$^+$ F4/80$^+$ cells
R14 CD45$^+$ Ly6G$^-$ CD11c$^-$ SiglecF$^-$ CD11d$^+$ F4/80$^+$ Ly6C^{high} cells (**Infl.Mo**)

Figure S1: Exemplary flow cytometric gating strategy for the analysis of innate immune cells. Representative dot plots illustrating the gating strategy of innate immune cells in BALC and lungs. alvM, Alveolar Macrophages; BALC, Bronchoalveolar lavage cells; Infl.Mo, Inflammatory Monocytes; matDCs, mature Dendritic Cells; PMNs, Polymorphonuclear Neutrophils.

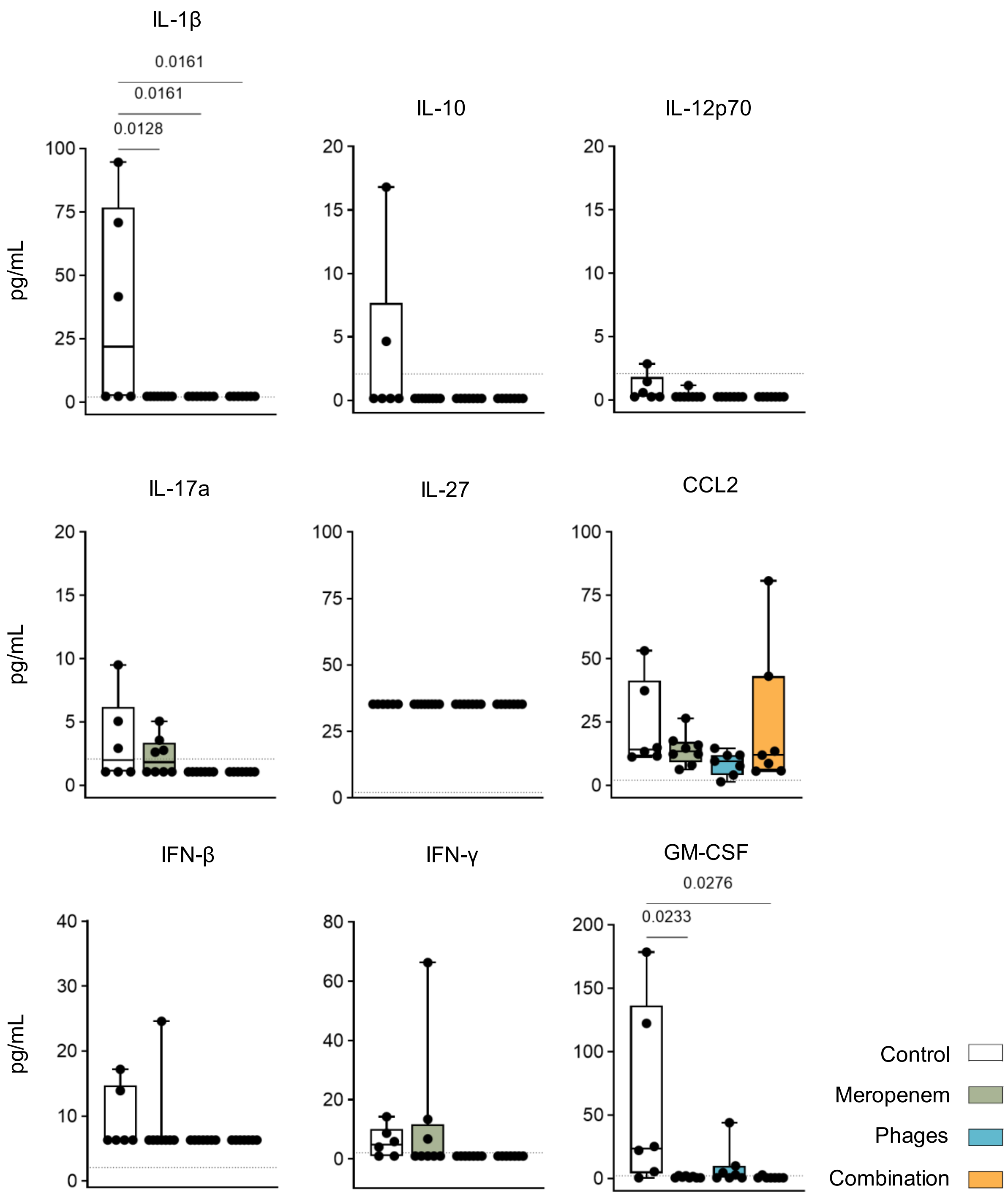

Figure S2: Inflammatory cytokines in BALF of mice with *Pseudomonas*-induced VAP. Level of pro-inflammatory cytokines (pg/mL) in BALF of mice with VAP analyzed at 24 hpi. Results are shown as box plots depicting median, quartiles, and range, as determined by ordinary one-way ANOVA with Tukey´s multiple comparisons test: *$p < 0.05$; **$p < 0.01$; $n = 6 – 8$ mice per group. (Control group n = 6; Meropenem group n = 8; Phages group n = 7; Combination group n = 7). Color coding for groups: Control group: white, Meropenem group: green, Phages group: blue; Combination group: orange. Dotted lines represent the detection limit, respectively. BALF, bronchoalveolar lavage fluid; IL, interleukin; CCL2, chemokine (C-C motif) ligand 2; GM-CSF, Granulocyte-macrophage- colony stimulating factor. Source data are provided as a Source Data file.

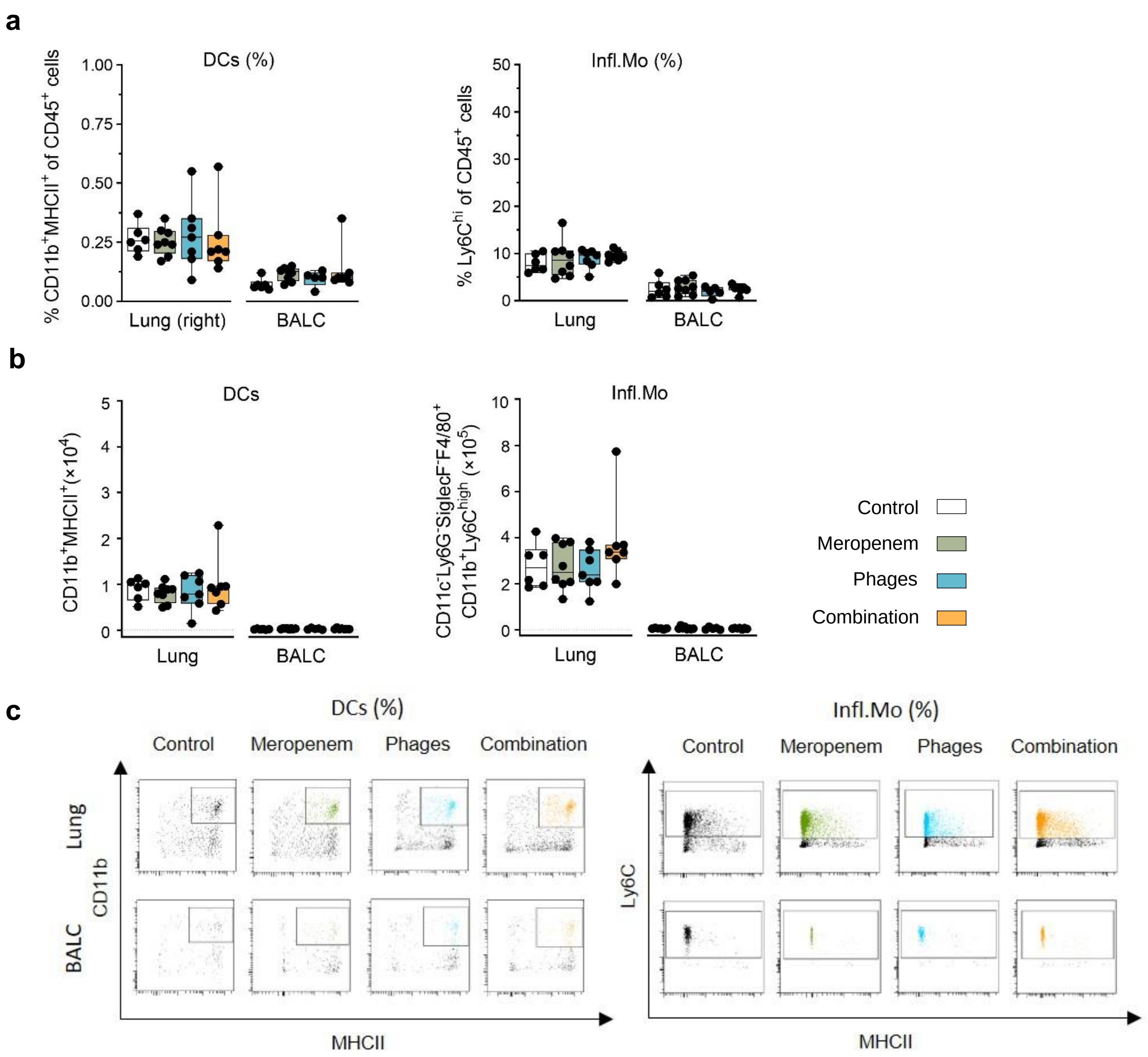

Figure S3: Phage treatment of *Pseudomonas*-induced VAP in mice had no impact on innate immune cells in BAL and lungs. Bar graphs depicting (**a**) the percentage, (**b**) total cell numbers or (**c**) representative dot plots of mature dendritic cells (DCs) and inflammatory monocytes (Infl.Mo) in BALC and half lungs (left) of mice with VAP analyzed at 24 hpi by flow cytometry. Results are shown as box plots depicting median, quartiles, and range, as determined by 2-way ANOVA with Tukey's multiple comparisons test; n = 6 – 8 mice per group. (Control group n = 6; Meropenem group n = 8; Phages group n = 7 (except Phages group BALC n = 5, technical loss of n = 2); Combination group n = 7). Color coding for groups: Control group: white, Meropenem group: green, Phages group: blue; Combination group: orange. For full gating strategy and cell type identifying markers see Supplementary Figure S1. BALC, bronchoalveolar lavage cells. Source data are provided as a Source Data file.

Supplementary Information

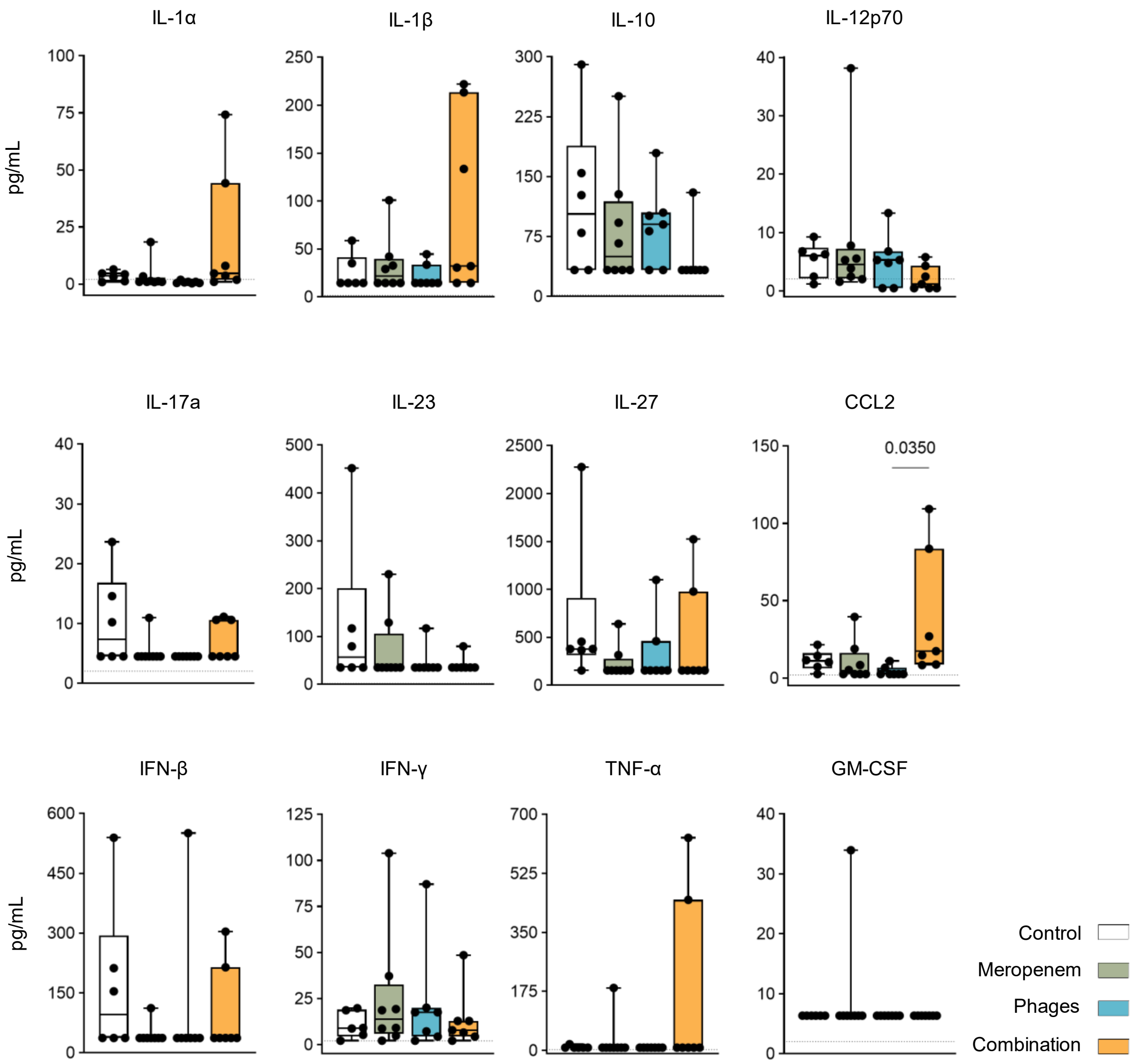

Figure S4: Inflammatory cytokines in plasma of mice with *Pseudomonas*-induced VAP. Level of pro-inflammatory cytokines (pg/mL) in plasma of mice with VAP analyzed at 24 hpi. Results are shown as box plots depicting median, quartiles, and range, as determined by ordinary one-way ANOVA with Tukey´s multiple comparisons test; *n* = 6 – 8 mice per group. (Control group n = 6; Meropenem group n = 8; Phages group n = 7; Combination group n = 7). Color coding for groups: Control group: white, Meropenem group: green, Phages group: blue; Combination group: orange. Dotted lines represent the detection limit, respectively. CCL2, chemokine (C-C motif) ligand 2; IL, interleukin; GM-CSF, Granulocyte-macrophage- colony stimulating factor. Source data are provided as a Source Data file.

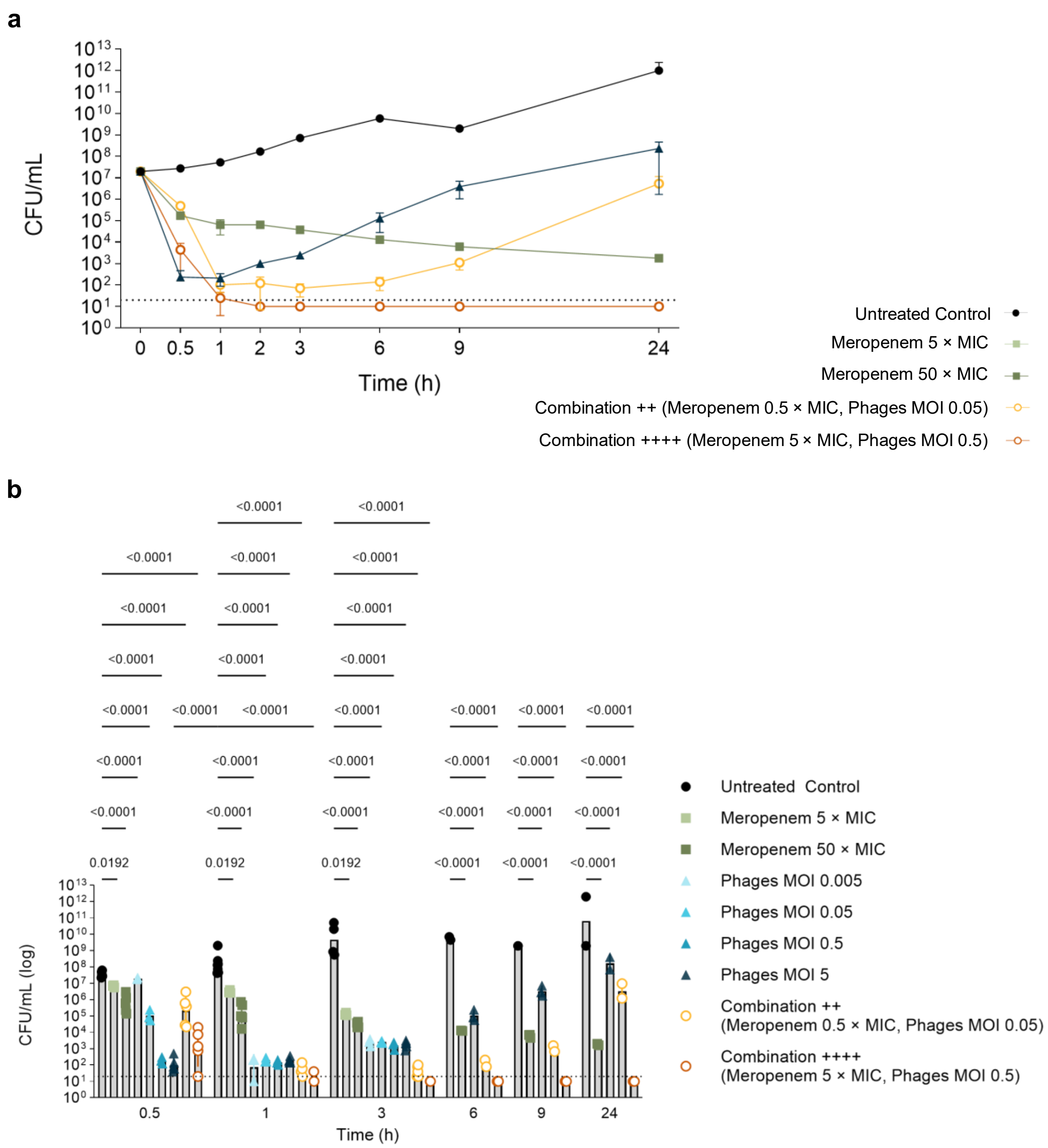

Figure S5: Long term analysis and direct comparison of the bacterial clearance in the Infection assay with different antibacterials. For the infection assay PAO1 were cultured in the absence (control) or presence of meropenem (MIC = 1 µg/mL), the *Pseudomonas*-specific phage cocktail, or the combination of both in different concentrations as indicated. (**a**) Colony-forming units (CFU/mL) according to time. Data are expressed as means of 2-3 independent experiments ± SD. Dotted line reflects detection limit. (**b**) Combined results of Fig 5b and Fig S5a are shown as means ± SD of bacterial load at different time points, as determined by two-way ANOVA with Dunett´s multiple comparisons test: *p < 0.05, **p < 0.01, ***p < 0.001, ****p < 0.0001; n = 3-6. Dotted line reflects detection limit. MIC, minimum inhibitory concentration; MOI, multiplicity of infection. Source data are provided as a Source Data file.

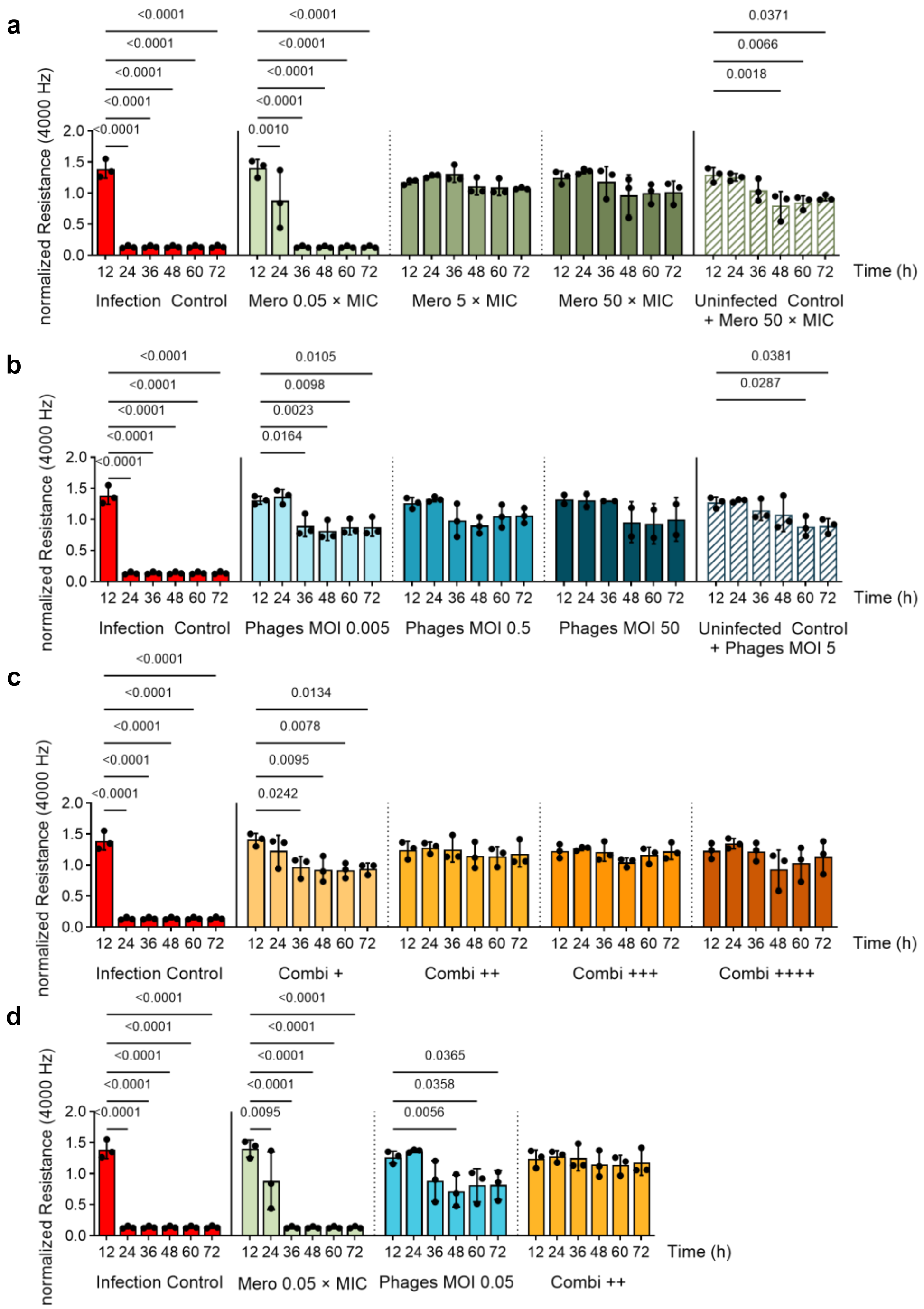

Figure S6: Loss of epithelial cell integrity in a dose dependent-manner by meropenem treatment of *P. aeruginosa*-infection *in vitro*. HPAEpiC monolayers in ECIS[TM] array wells were washed with antibiotic-free medium followed by infection with PAO1 (MOI 1) and treatment with (**a**) meropenem (Mero MIC = 1 µg/mL), (**b**) the Pseudomonas-specific phage cocktail (Phages), or (**c**) the combination of both (Combi +: Mero 0.005 × MIC, Phages MOI 0.005; Combi ++: Mero 0.05 × MIC, Phages MOI 0.05; Combi +++: Mero 0.5 × MIC, Phages MOI 0.5; Combi ++++: Mero 5 × MIC, Phages MOI 0.5) for 72 h. Cell toxicity controls were assessed by treating HPAEpiC with high dose of Meropenem (50 × MIC) or Phages (MOI 5) without infection. (**d**) Direct comparison of ECIS experiments with combination and single therapy. Results are shown as means ± SD of normalized resistance (4000 Hz) at different time points, as determined by two-way ANOVA with Tukey´s multiple comparisons test: *p < 0,05, **p < 0,01, ***p < 0,001, ****p < 0,0001; (a - d) *n* = 3 independent experiments, while each condition was run in triplicates per experiment, except (b) *n* = 2 for Phages MOI 50. Infection control (PAO1 MOI 1) same dataset in each figure. ECIS, electric cell-substrate impedance sensing; HPAEpiC, human primary alveolar epithelial cells; MIC, minimum inhibitory concentration. Source data are provided as a Source Data file.

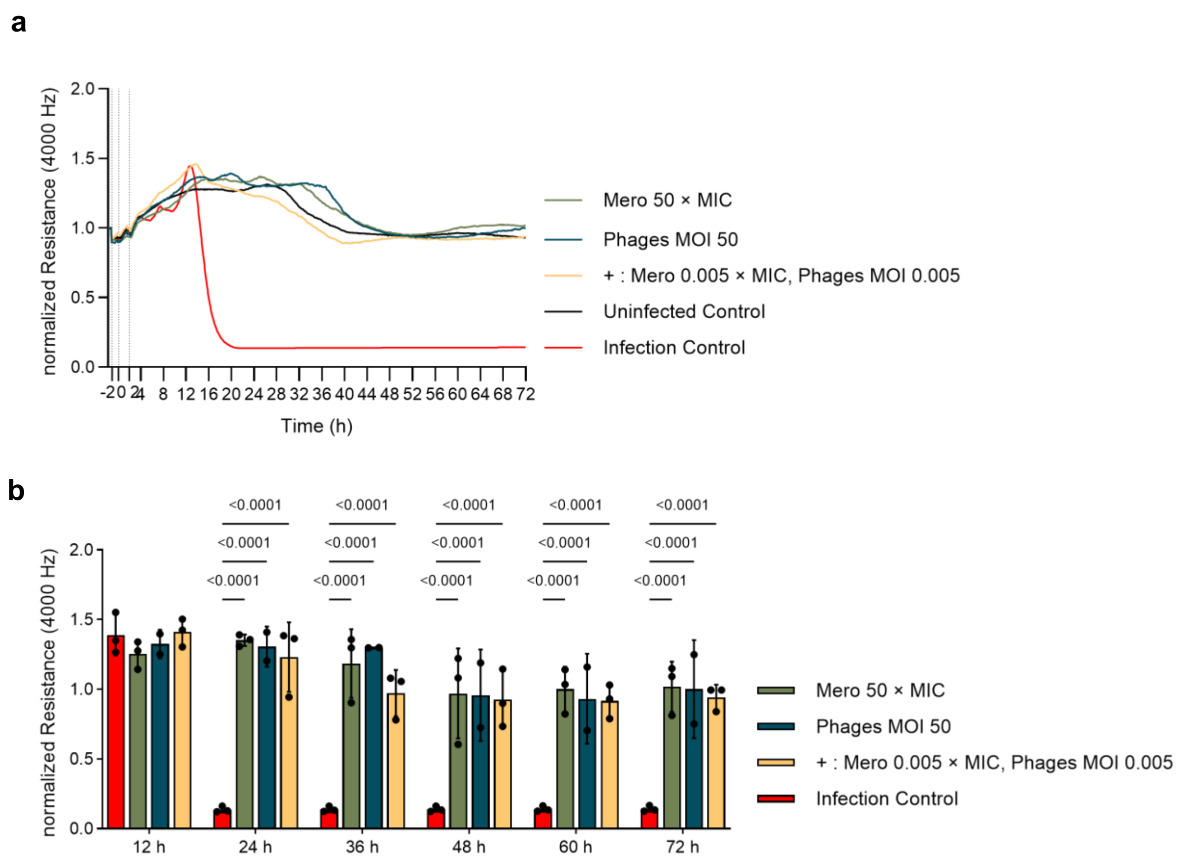

Figure S7: Adjunctive phage therapy against *Pseudomonas*-infection *in vitro* as successful as high-dose monotherapy. HPAEpiC monolayers in ECIS^{TM} array wells were washed with antibiotic-free medium followed by infection with PAO1 (MOI 1) and treatment with (**a**) meropenem (Mero MIC = 1 µg/mL), the Pseudomonas-specific phage cocktail (Phages), or the combination of both (Combi +: Mero 0.005 × MIC, Phages MOI 0.005) for 72 h. (**b**) Direct comparison of ECIS experiments with combination and single therapy. Results are shown as means ± SD of normalized resistance (4000 Hz) at different time points, as determined by two-way ANOVA with Tukey´s multiple comparisons test: *p < 0.05, **p < 0.01, ***p < 0.001, ****p < 0.0001; *n* = 3 independent experiments, while each condition was run in triplicates per experiment. Infection control (PAO1 MOI 1) same dataset in each figure. Uninfected Control are only HPAEpiC. ECIS, electric cell-substrate impedance sensing; HPAEpiC, human primary alveolar epithelial cells; MIC, minimum inhibitory concentration. Source data are provided as a Source Data file.

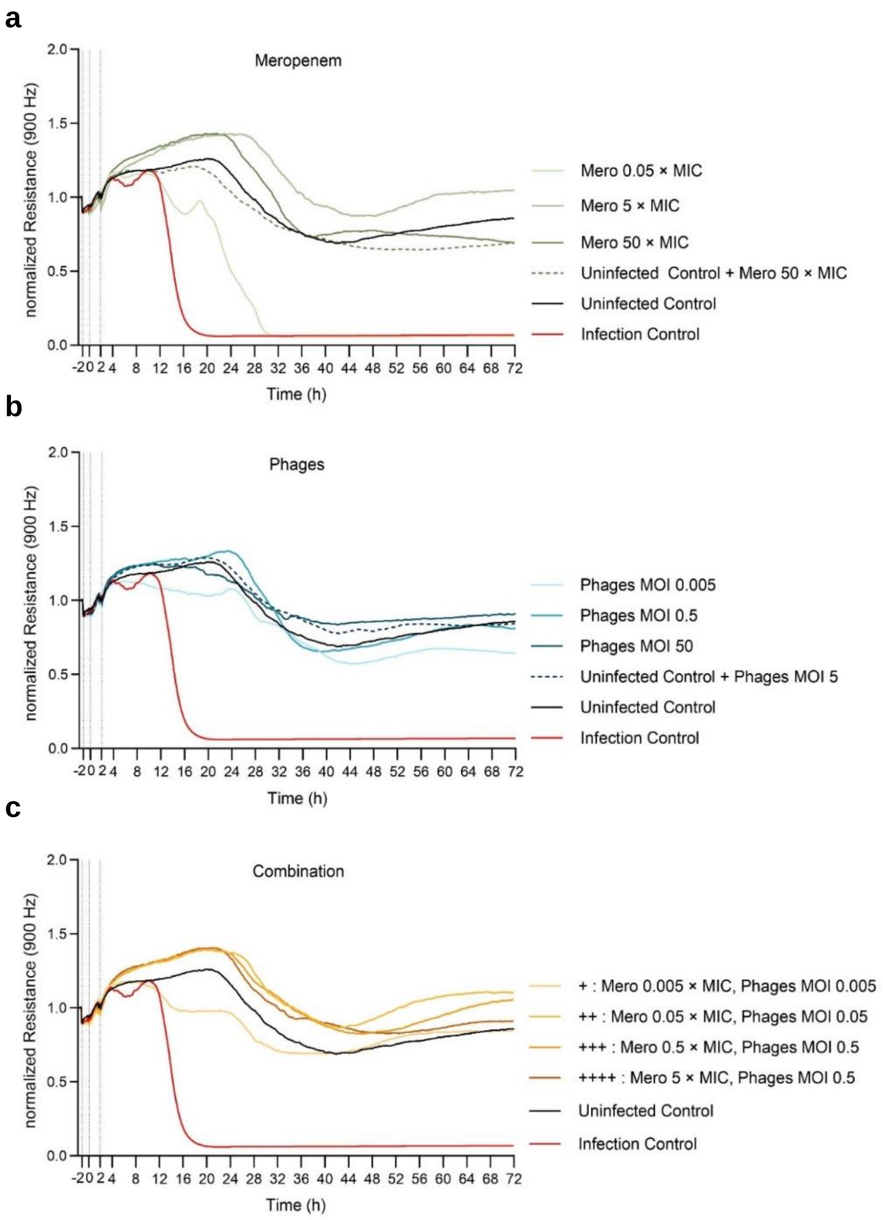

Figure S8: Improved lung epithelial cell integrity by phage treatment of bacterial infection *in vitro*. Measuring the transepithelial electric resistance (TEER) of epithelial cell monolayers by using ECIS^{TM} system upon infection with PAO1 (MOI 1) and subsequent treatment with (**a**) meropenem (Mero; MIC = 1 µg/mL), (**b**) the *Pseudomonas*-specific phage cocktail (Phages), or (**c**) a combination of both in indicated concentrations for 72 h. Means of normalized resistance (900 Hz) versus time curves are shown; each isolate was run in 3 independent experiments while each condition where run in triplicates (96W10idf). Dotted line indicates the starting time of starving (-2 h), infection (0 h) and treatment (2 h). Uninfected Control and Infection Control (PAO1 MOI 1) same dataset in each figure. Uninfected Control are only HPAEpiC. ECIS, electric cell-substrate impedance sensing; HPAEpiC, human primary alveolar epithelial cells; MIC, minimum inhibitory concentration. Source data are provided as a Source Data file.
