# Supplementary material for: Adjunctive phage therapy improves antibiotic treatment of ventilator-associated-pneumonia with Pseudomonas aeruginosa
Source: Nat Commun. 2025 May 15;16:4500. doi: 10.1038/s41467-025-59806-y (PMC12078490; doi:10.1038/s41467-025-59806-y)
Supplement: Supplementary file 2 — Reporting Summary [file 41467_2025_59806_MOESM2_ESM.pdf]

nature portfolio

Corresponding author(s): Chantal Weissfuss, Geraldine Nouailles

Last updated by author(s): Apr 6, 2025

Reporting Summary

Nature Portfolio wishes to improve the reproducibility of the work that we publish. This form provides structure for consistency and transparency in reporting. For further information on Nature Portfolio policies, see our Editorial Policies and the Editorial Policy Checklist.

Statistics

For all statistical analyses, confirm that the following items are present in the figure legend, table legend, main text, or Methods section.

n/a	Confirmed	
☐	☒	The exact sample size (*n*) for each experimental group/condition, given as a discrete number and unit of measurement
☐	☒	A statement on whether measurements were taken from distinct samples or whether the same sample was measured repeatedly
☐	☒	The statistical test(s) used AND whether they are one- or two-sided<br>*Only common tests should be described solely by name; describe more complex techniques in the Methods section.*
☐	☒	A description of all covariates tested
☐	☒	A description of any assumptions or corrections, such as tests of normality and adjustment for multiple comparisons
☐	☒	A full description of the statistical parameters including central tendency (e.g. means) or other basic estimates (e.g. regression coefficient) AND variation (e.g. standard deviation) or associated estimates of uncertainty (e.g. confidence intervals)
☐	☒	For null hypothesis testing, the test statistic (e.g. *F*, *t*, *r*) with confidence intervals, effect sizes, degrees of freedom and *P* value noted<br>*Give P values as exact values whenever suitable.*
☒	☐	For Bayesian analysis, information on the choice of priors and Markov chain Monte Carlo settings
☒	☐	For hierarchical and complex designs, identification of the appropriate level for tests and full reporting of outcomes
☒	☐	Estimates of effect sizes (e.g. Cohen's *d*, Pearson's *r*), indicating how they were calculated

Our web collection on statistics for biologists contains articles on many of the points above.

Software and code

Policy information about availability of computer code

Data collection	BD FACSDiva 8.0.1 (FC analysis); LEGENDplexTM Software (Multiplex ELISA); SkanIt Software for Microplate Readers RE, ver 7.0.1.4; Applied BioPhysics - ECIS Software v1.2.254.0. PC (ECIS Z-Theta)
Data analysis	Data were analyzed using GraphPad Prism 10 (San Diego, CA, USA).

For manuscripts utilizing custom algorithms or software that are central to the research but not yet described in published literature, software must be made available to editors and reviewers. We strongly encourage code deposition in a community repository (e.g. GitHub). See the Nature Portfolio guidelines for submitting code & software for further information.

Data

Policy information about availability of data

All manuscripts must include a data availability statement. This statement should provide the following information, where applicable:

- Accession codes, unique identifiers, or web links for publicly available datasets
- A description of any restrictions on data availability
- For clinical datasets or third party data, please ensure that the statement adheres to our policy

Source data are provided with this paper. The experimental murine in vivo and human in vitro data generated in this study are provided in the Supplementary Information and in the Source Data file.

Research involving human participants, their data, or biological material

Policy information about studies with human participants or human data. See also policy information about sex, gender (identity/presentation), and sexual orientation and race, ethnicity and racism.

Reporting on sex and gender	n/a
Reporting on race, ethnicity, or other socially relevant groupings	n/a
Population characteristics	n/a
Recruitment	n/a
Ethics oversight	n/a

Note that full information on the approval of the study protocol must also be provided in the manuscript.

Field-specific reporting

Please select the one below that is the best fit for your research. If you are not sure, read the appropriate sections before making your selection.

☒ Life sciences ☐ Behavioural & social sciences ☐ Ecological, evolutionary & environmental sciences

For a reference copy of the document with all sections, see nature.com/documents/nr-reporting-summary-flat.pdf

Life sciences study design

All studies must disclose on these points even when the disclosure is negative.

Sample size	Sample sizes of individual groups as well as biological and technical replicates are indicated in the figure legends. Animal group sizes are n = 6-8. Sample size selection for animal experiments followed the 3R principles and was based on experience on the given model system. Minimal animal number required to obtain interpretable data. In vitro experiments were repeated in at least triplicate to ensure reproducibility.
Data exclusions	No data was excluded in the analysis.
Replication	Variability between replicates of in vivo or in vitro experiments are displayed in the figures by showing standard deviations (SD) or standard error of mean (SEM) or box plots depicting median, quartiles, and range.
Randomization	Animals were assigned randomly to experimental groups. For in vitro experiments, conditions were not assigned randomly, but by location on well plates, same group in proximity to avoid experimental errors during individual treatments.
Blinding	For the animal experiments blinding was not possible as the animals received different treatments, and, per regulations, treatment details had to be clearly displayed on the cage cards, making them accessible to the experimenters at all times.<br>All mice were subjected to pre-defined score sheets and clinical disease score.<br>In vitro experiments were not blinded as data was collected and in part analysed during perfomance of experiment and investigator needed to know how to treat individual groups.

Reporting for specific materials, systems and methods

We require information from authors about some types of materials, experimental systems and methods used in many studies. Here, indicate whether each material, system or method listed is relevant to your study. If you are not sure if a list item applies to your research, read the appropriate section before selecting a response.

Materials & experimental systems

n/a	Involved in the study
☐	☒ Antibodies
☐	☒ Eukaryotic cell lines
☒	☐ Palaeontology and archaeology
☐	☒ Animals and other organisms
☒	☐ Clinical data
☒	☐ Dual use research of concern
☒	☐ Plants

Methods

n/a	Involved in the study
☒	☐ ChIP-seq
☐	☒ Flow cytometry
☒	☐ MRI-based neuroimaging

Antibodies

Antibodies used	For flow cytometry analysis, BAL and lung cells were blocked with anti-CD16/CD32 (2.4G2; cat. nr. 553142; BD, Heidelberg, Germany) and stained with anti-CD45 (30F11; cat. nr. 103108; BD, Heidelberg, Germany), anti-CD11c (HL3; cat. nr. 550261; BD, Heidelberg, Germany), anti-CD11b (M1/70; cat. nr. 25-0112-82; eBioscience, Frankfurt, Germany), anti-F4/80 (BM8; cat. nr. 12-4801-80; eBioscience, Frankfurt, Germany), anti-Ly6G (1A8; cat. nr. 560602; BD, Heidelberg, Germany), anti-LyGC (HK.1.4; cat. nr. 128033; BioLegend, San Die-go, USA), anti-MHCII (M5/114.15.2; cat. nr. 56-5321-82; eBioscience, Frankfurt, Germany), or anti-Siglec F (E502440; cat. nr. 562681; BD, Heidelberg, Germany) monoclonal antibodies (mAbs).
Validation	All antibodies/clones are commonly used in flow cytoemtry with well established performance. Antibodies were validated by their manufacturers. Validation statements as well as further information and Certificates of Analysis of each antibody can be found regularly on the websites of BD, eBioscience and BioLegend.

Eukaryotic cell lines

Policy information about cell lines and Sex and Gender in Research

Cell line source(s)	Human primary alveolar epithelial cells (HPAEpiC) from human lung tissue were purchased from Cell Biologics (H-6053; Chicago, Illinois, USA).
Authentication	Human Primary Alveolar Epithelial Cells from Cell Biologics are tested for expression of markers using antibody, E-cadherin (Catalog No. 610182 from BD) or ZO-1 Rabbit Polyclonal Antibody (Catalog No. 617300; from Life Technologies) by immunofluorescence staining. (Statement of CellBiologics)
Mycoplasma contamination	All cells test negative for mycoplasma, bacteria, yeast, and fungi. HIV-1, hepatitis B and hepatitis C are not detected for all donors and/or cell lots. (Statement of CellBiologics)
Commonly misidentified lines (See ICLAC register)	No commonly misidentified cell line was used.

Animals and other research organisms

Policy information about studies involving animals; ARRIVE guidelines recommended for reporting animal research, and Sex and Gender in Research

Laboratory animals	C57BL/6J WT mice (Charles River, Sulzfeld, Germany), female, 8 to 10-week old
Wild animals	The study did not involve wild animals.
Reporting on sex	We used female C57BL/6J WT mice to minimize variability in pneumonia severity due to sex differences and have stated this in the manuscript.
Field-collected samples	The study did not contain field-collected samples.
Ethics oversight	Landesamt für Gesundheit und Soziales (LAGeSo), Berlin, Germany<br>Tierschutzbeauftragte, Charité - Universitätsmedizin Berlin, Germany

Note that full information on the approval of the study protocol must also be provided in the manuscript.

Plants

Seed stocks	n/a
Novel plant genotypes	n/a
Authentication	n/a

Flow Cytometry

Plots

Confirm that:

☒ The axis labels state the marker and fluorochrome used (e.g. CD4-FITC).

☒ The axis scales are clearly visible. Include numbers along axes only for bottom left plot of group (a 'group' is an analysis of identical markers).

☒ All plots are contour plots with outliers or pseudocolor plots.

☒ A numerical value for number of cells or percentage (with statistics) is provided.

Methodology

Sample preparation	Sample preparation (i. e. Leukocyte Differentiation in BAL and Lungs by Flow Cytometry) is explained in the method section in detail.
Instrument	BD FACSCantoTM II, BD, Heidelberg, Germany
Software	BD FACSDiva 8.0.1
Cell population abundance	Typically samples were measured using at least 100,000 CD45+ cells (lungs) or, when not reachable due to small cell populations, all cells (BAL).
Gating strategy	The Gating Strategy for the flow cytometry analysis of innate immune cells in murine lungs and BAL are explained in the Supplementary Figure S1 in detail.

☒ Tick this box to confirm that a figure exemplifying the gating strategy is provided in the Supplementary Information.
